# Air quality in the operating room: Surgical site infections, HVAC systems and discipline – position paper of the German Society of Hospital Hygiene (DGKH)

**DOI:** 10.3205/dgkh000335

**Published:** 2019-12-04

**Authors:** Walter Popp, Christof Alefelder, Sonja Bauer, Georg Daeschlein, Petra Geistberger, Sabine Gleich, Caroline Herr, Nils-Olaf Hübner, Lutz Jatzwauk, Wolfgang Kohnen, Rüdiger Külpmann, Friederike Lemm, Barbara Loczenski, Jörg Spors, Peter Walger, Markus Wehrl, Klaus-Dieter Zastrow, Martin Exner

**Affiliations:** 1 German Society for Hospital Hygiene (DGKH), Berlin, Germany

**Keywords:** operating room, ventilation, laminar airflow, LAF, surgical site infection, discipline

## Abstract

In recent years, there has been an ongoing discussion about the value of laminar airflow (LAF=low turbulence displacement ventilation) in the operating room for prevention of surgical site infections (SSI). Some publications, e.g., from the WHO, issued the demand to no longer build LAF ceilings in operating rooms.

The present statement deals critically with this position and justifies the use of LAF ceilings in different ways:

- Many of the papers cited by the WHO and others for the case against LAF do not provide reliable data.
- The remaining studies which might be used for answering the question give quite different results, also in favor of LAF.
- The size of the LAF ceiling in many studies is not given or mostly too small in comparison to actual technical requirements.
- LAF in different countries can mean quite different techniques (e.g., the US in comparison to Germany) so that the results of studies that do not take this into account may not be comparable.
- LAF has positive effects in terms of reducing particulate and bacterial load, associated with increased airflow in the surgical working area. A reduction of carcinogenic substances in the air may also be assumed, which would increase workers’ safety.

Thus, this paper recommends building LAF ceilings in the future as well, depending on the operations intended.

Further, this paper gives an overview of possible reasons for surgical site infections and highlights the importance of discipline in the operating theatre.

## Zusammenfassung

In den letzten Jahren wird fortwährend über den infektionspräventiven Wert von laminar airflow (LAF=Turbulenz-arme Verdrängungsströmung) im Operationssaal diskutiert. Einige Publikationen, z.B. von der WHO, fordern, keine LAF-Decken in Operationssälen mehr einzubauen.

Die Stellungnahme setzt sich kritisch mit dieser Position auseinander und begründet den Einsatz von LAF-Decken auf unterschiedliche Weise:

- Viele der zitierten Artikel zu LAF sind bezüglich der Datenqualität nicht verlässlich.
- Die verbleibenden Studien, die zur Beantwortung der Frage herangezogen werden können, ergeben für LAF unterschiedliche Ergebnisse.
- Die Größe der LAF-Decken ist in vielen Studien nicht angegeben bzw. im Vergleich zu den tatsächlichen technischen Anforderungen meist zu klein.
- LAF kann in verschiedenen Ländern sehr unterschiedliche Techniken beinhalten (z.B. USA im Vergleich zu Deutschland), so dass die Ergebnisse von Studien, die das nicht erfassen, nicht vergleichbar sind.
- LAF hat positive Auswirkungen hinsichtlich der Reduktion von Partikeln und Mikroorganismen in der Luft im Bereich des OP-Felds, verbunden mit einem erhöhten Luftvolumenstrom. Daraus kann zugleich eine Reduktion kanzerogenen Substanzen in der Luft abgeleitet werden und damit ein besserer Arbeitsschutz.

In der Konsequenz wird empfohlen, in Abhängigkeit von den beabsichtigten Operationen auch in Zukunft LAF-Decken zu bauen.

Der Artikel gibt zugleich einen Überblick darüber, welche Ursachen für postoperative Wundinfektionen in Frage kommen und weist auf die Bedeutung der Disziplin im OP hin.

## A brief history of general heating, ventilation, and air conditioning (HVAC)

Until the late 1970s, little attention was paid to the impact of ventilation systems on air quality. 

It was only in the wake of systematic investigations of, e.g., the source of *Legionella pneumophila* and the cause of “sick building syndrome” [1] that relatively rapid progress was made. For example, in northern Europe in particular, standards and guidelines were enacted on hygienic planning, implementation and operation of HVAC systems. Since 1999, personnel working with HVAC systems in Germany must be trained by certified bodies [2].

In Germany, a conventional HVAC system, such as is used both in offices and in hospitals, must be manufactured in accordance with VDI 6022. Among other things, this calls for an F7 filter (as per the new ISO standard 16890 minimum filtration efficiency ISO ePM2.5 >65%) at the inlet to the main unit and an F9 filter (as per the new ISO standard 16890 minimum filtration efficiency ISO ePM1 >80%) at the supply air outlet of the main unit.

## The history of heating, ventilation, and air conditioning in the operating room

For centuries, the air has been viewed as the main route of transmission of infectious diseases.

During the 1950s, the principle pathogen reservoirs for surgical site infections (SSIs) were thought to be the nasopharyngeal region of the surgical team and the operating room air [3].

In the 1960s, the first isolated studies [4] were carried out on the hygienic impact of ventilation concepts.

In the 1980s, Lidwell et al. [5], [6], [7], [8], [9], [10], [11], [12] published various studies reporting an approximately 2-fold reduction in deep SSIs after total hip or knee replacement operations when LAF ventilation was used compared with conventional ventilation. The infection rate was further reduced when body-exhaust suits were additionally worn (around 4.5-fold). The reduction was 3- to 4-fold when perioperative antibiotics were administered; according to Lidwell et al., the effects of the air and antibiotic administration were additive and independent of each other [9], [11].

Likewise during the 1980s, Rüden et al. demonstrated that septic operations were not associated with increased airborne microbial counts [3].

In 2001, in a review of the literature conducted on behalf of the DGKH, Kappstein reported that airborne pathogens present as droplet nuclei could only originate from the nasopharyngeal region and dead skin of surgical personnel. On using HVAC systems with turbulent mixed airflow, bacteria could be spread from persons at the periphery of the operating room to the wound. Therefore, HVAC systems would have to supply the area around the surgical field and instrument table with air with only a low microbial count. The airflow principle would have to be stable LAF (low-turbulence displacement) ventilation [3].

Modern HVAC systems in the operating room have the following main functions:

- Contribute to patient protection and should therefore assure air of the highest hygiene quality (with only a low microbial count or even sterile air).
- Provide thermal comfort for operating room personnel
- Remove harmful particles, e.g., carcinogenic surgical smoke gases or anaesthesia gases
- Meet technical process or product requirements (functionality and safety).

Modern HVAC systems as used in the operating room have three filtration stages; in Germany the third stage usually consists of a terminal filter mounted flush with the ceiling in the operating room. This filtration stage should be of H13 or H14 quality (pursuant to DIN EN 1822). For LAF ventilation (low turbulence displacement ventilation), a fabric ceiling panel measuring approx. 3.2x3.2 m and fitted over a large area with the terminal filters is currently required. The low turbulence is ensured by the fabric. For flawless functioning of the ventilating ceiling, the incoming air must be somewhat cooler than the ambient air so that it will slowly drop downwards in accordance with physical principles. The latter are effective without further interventions through utilization of the operating room and the heat input from equipment and persons.

LAF ventilation has long been the gold standard for operating rooms. With the introduction of DIN 1946-4 in 2008, it became mandatory for a number of operations, something that was difficult to comprehend in certain respects and led to considerable criticism. In 2008, the Commission for Hospital Hygiene and Infection Prevention at the Robert Koch Institute (*KRINKO*) adopted a critical stance towards this standard [13], stating that in view of study findings, there was no justification for differentiation into “Class Ia” (LAF) and “Class Ib” rooms (LAF ceiling or turbulent air).

An amended version of DIN 1946-4 was published in 2018 [14], setting out that the Class Ia room continued to be justified and that the type of surgery with the most stringent requirements defined the room class of an operating room. But that meant that the Class Ia room continued to be the state of the art and would be taken into account in the building design. 

## Verification of HVAC systems

The design of the LAF ventilation systems (low-turbulence displacement ventilation) long used in the pharmaceutical and electrical industries has improved considerably since the early 2000s for the creation of a clean zone in operating rooms.

Since the early 2000s, the German-speaking, Dutch and Scandinavian standards and guidelines have featured comprehensive test methods for verification of the impact of LAF and mixed airflow concepts on the air quality in operating rooms. For example, when using LAF, the introduction of particles (to which pathogens can adhere during surgery) must at least a factor of 100 lower than for mixed airflow. To that end, the degree of protection must be ascertained in accordance with precise specifications for the surgical field with swivel and surgical light fixtures (degree of protection >2). In contrast, for mixed airflow, it is more practical to determine the recovery time (<20 min) of a particle burden in the room after elimination of the particle source through continuous dilution of the ambient air with the inflow of sterile filtered air. With LAF, the recovery time in the protected area would be less than 2 min because of its directed airflow.

## Current objections to LAF

In 2016/2017, Bischoff et al. [15], Allegranzi et al. [16] and the WHO [17] published papers objecting to the installation of LAF ventilation. The studies [15], [16] on which the publications were based reported inconsistent SSI rates for total hip and knee arthroplasties and other operations in relation to the ventilation concept. The study by Brandt et al. [18] played an important role in the past, because for diverse operations with the exception of colon surgery, it was unable to find any evidence that LAF had a protective effect; in fact, it predominantly identified increased infection risks. For surgery performed using LAF, higher SSI rates were determined for total hip arthroplasty (sign.), total knee arthroplasty, appendectomy, cholecystectomy and hernia operations, but in contrast, fewer infections for colon surgery. The study included data from the German National Nosocomial Infection Surveillance System (*KISS*) from 2000 to 2004 (99,230 operations with 1901 SSIs corresponding to a rate of 1.9%). In August 2004, i.e., after data collection, a questionnaire was sent out to the respective infection control teams to obtain information on the HVAC systems. The publication did not include any critical discussion of the findings; in particular, the reasons why LAF should yield better results for colon surgery were not explored. 

Assadian et al. [19] and Kramer et al. [20] addressed problems with that study. The latter stated that the technical parameters and configuration of the HVAC systems had not been checked at the start of the survey. Furthermore, it was likely that the requisite ceiling panel of 3.2x3.2 m was not available in the majority of cases, since the DIN standard 1946-4 first described that ventilation concept only in December 2008. It was thought that several potential confounders had not been considered, for example, the surgeons, operating room furnishings, surgical drapes, patient risk factors, perioperative prophylaxis, hair removal or follow-up. Moreover, the postal survey had critical limitations, since even the technical staff were often not able to give proper responses regarding the type of ventilation in use, e.g., classifying perforated sheet ceilings as LAF ceilings. 

Another critical aspect is that, especially in the case of total prothesis implantations, the majority of SSIs only manifest after patient discharge from the hospital, i.e., at a time not properly covered by the KISS method. Data from Switzerland [21], [22] demonstrate that around 80% of SSIs in total hip arthroplasty, and 95% of SSIs in total knee arthroplasty occur after hospital discharge. That means that for the knee arthroplasty rates recorded only on an in-patient basis, the actual SSI rates are 20 times higher. A systematic literature search by Woelber et al. [23] showed that 60% of nosocomial infections manifest after discharge from the hospital.

Furthermore, there is doubt about the quality of hospital-based (in-patient) recording of SSI rates as done with the *KISS* module. In a group of 1,215 patients in Sweden [24], healthcare-associated (HAI) /nosocomial infections were diagnosed by the hospital’s infection control team and in parallel by external experts, citing rates of 9.3% and 13.1%, respectively. Differences in the quality of data collection were also identified when comparing the Swiss findings (Swissnoso) with the German data (*KISS* and external quality assurance). Some examples of HAI rates are given in Table 1 (Tab. 1).

Hence, in most cases, Switzerland was found to have 2 to 3 times more postoperative SSIs than Germany (*KISS*), whereas the findings by the external quality assurance in Germany for Caesarean sections and total hip and knee arthroplasties were around 2 to 4 times less than the *KISS* rates. Since it is unlikely that the Swiss healthcare system is poorer that its German counterpart, the differences must be due to methodical disparities, e.g., data recorded for variable periods after patient discharge (in Switzerland, there are outcome data for 90% of patients recorded even one year after hospital stay). It must therefore be assumed that in the study by Brandt et al. [18], the reported infection rates were underestimated.

The criticism levelled at the study by Brandt et al. [18] led to a follow-up study by Breier et al. [25]. This retrospective cohort study based on data from the *KISS* data pool included 33,463 total hip arthroplasties and 20,554 total knee arthroplasties covering the years 2004 to 2009. That was followed in 2009 by an online survey when hospitals were also asked about the size of the LAF ceiling panel. It was revealed that only 35–40% of operating rooms had an LAF ceiling measuring at least 3.2x3.2 m. Again, some of the operations performed using LAF had higher infection rates, but these were markedly lower than in the study by Brandt et al. [18]. For total knee arthroplasties, LAF was even found to have a protective effect, although this was not significant. One limitation cited was that – as in the study by Brandt et al. [18] – data on perioperative antibiotic prophylaxis were not recorded and post-discharge data were not “systematically” recorded with the *KISS* method. Hence, the SSI rates were far lower than in the study by Brandt et al. [18], or LAF was even found to have a protective effect for total knee arthroplasties. Besides, further limitations were identified for the investigation method (inadequate post-discharge data collection, perioperative antibiotic prophylaxis [PAP] not taken into account, ceiling panels too small in most operating rooms), so that the study cannot be used as a valid basis for assessment of LAF ceilings with the current standard dimensions of 3.2x3.2 m.

Another problem is the lack of standardized qualification of LAF systems prior to 2008. The methods for operating room qualification were first published in 2008 with the introduction of DIN 1946-4. Therefore, reliable functionality of LAF systems before 2008 cannot be assumed.

It must be pointed out that in the German-speaking countries, the greatest changes in the design of operating room ventilation systems for Class Ia rooms were ushered in only as of 2002 and 2008, with the introduction of new standards in Switzerland (SWKI 99-3:2002) [26], Austria (ÖNORM H 6020:2007) [27], and Germany (DIN 1946-4:2008) [14]. Only in the aftermath of these publications has a minimum size 3.2x3.2 meters been required for LAF ceiling panels; furthermore, the test regulations for acceptance of LAF ceilings have been greatly tightened and brought into line with real working conditions. Until then, it was only possible to install much smaller ceiling panels (e.g., 1.8x2.4 m), which had a considerably reduced air flow rate.

Various critical remarks should be made about the studies evaluated by Bischoff et al. [15], while raising the following issues:

- While the cohort study by Kakwani et al. [28] (2007; UK) with a total of 435 patients was small, it demonstrated a significant reduction of the hemiarthroplasty infection rate from 5.8% to 1.4% upon using LAF. A particularly positive aspect of the study was that the follow-up period was at least one year.
- The study by Hooper et al. [29] (2011; New Zealand) is an evaluation of the New Zealand Joint Registry, showing higher infection rates with LAF, but the panel sizes were not reported. However, because only “early infections” were reported it is probable (see above) that these were greatly underreported. For example, for 51,485 total hip arthroplasties only 46 infections and for 36,826 total knee arthroplasties 50 infections were diagnosed. That corresponds to 0.09% and 0.14%, respectively. Hence, the rate for total hip arthroplasties at least was markedly lower than the values identified by the German external quality assurance team at 0.32% (IQTIQ 2017), which themselves have little validity and no doubt underestimate infection rates (see above). The data of the New Zealand Joint Registry are therefore not plausible and should not be used for evaluation of LAF ceiling panels.
- The study by Pedersen et al. [30] (2010; Denmark) is an evaluation of data from the Danish Hip Arthroplasty Registry for the years 1995 to 2008. The mean follow-up period was five years (range: 0 to 14 years). With 80,756 operations, there were 597 infections (a rate of 0.7%. With LAF, there were fewer infections (crude risk ratio [RR] 0.81, adjusted RR 0.90), but the differences were not significant.
- Similarly, the study by Namba et al. [31] (2012; USA) evaluated data from the Kaiser Permanente Total Joint Replacement Registry, with a reported follow-up period of one year. From the 30,491 total hip arthroplasties carried out between 2001 and 2009, there were 155 infections (a rate of 0.51%). The hazard ratio with LAF at 1.08 was not significant. As pointed out below, LAF in the USA is not necessarily comparable with LAF ventilation as used in Germany.
- Dale et al. [32] (2009; Norway) published a study on hip arthroplasty infections based on data from the Norwegian Arthroplasty Registry for the period 1987 to 2007. Out of 97,344 cases, there were 614 infections (a rate of 0.6%). The follow-up period continued up to the time of patient death, relocation or the end of 2007, with a range of 0 to 20 years. LAF was associated with a significantly increased risk with RR of 1.3 (p=0.006).
- Bosanquet et al. [33] (2013; UK) published a retrospective evaluation of a “single consultant”, over a period of one year, who investigated SSIs in 170 vascular patients. There were 23 SSIs (rate: 13.5%). With LAF, there were fewer infections –11% vs. 33% (p=0.034).
- The study by Jeong et al. [34] (2013; Korea) was a cohort study of gastric surgery in 10 hospitals with 2091 patients. The follow-up period was one month. There were 71 SSIs (rate: 3.4%), and the rates for individual hospitals were between 0 and 15.7%. Overall, there were fewer infections with LAF at 7.2% compared with 36.6% (significant). The major differences in SSI rates among the various hospitals are very conspicuous. Furthermore, data were collected separately on LAF and HEPA filters, suggesting that LAF in Korea need not necessarily be the same as that in Germany.
- Miner et al. [35] (2007; USA) investigated the rate of deep SSIs following 8288 total knee arthroplasties in 256 hospitals based on Medicare claims. Using LAF, an RR of 1.57 was calculated (not significant).
- Song et al. [36] (2012; Korea) conducted a retrospective cohort study in 26 hospitals between 2006 and 2009, recording SSIs after total hip and knee arthroplasties. Here a distinction was made between operations performed under LAF, operations with HEPA filter alone and operations with no mechanical ventilation. LAF was used as the reference. For the other two ventilation types, increased risks were seen in most cases, and were significant for total knee arthroplasties conducted in operating rooms with HEPA filter alone, with odds ratio (OR) of 1.83.

The studies included in a meta-analysis by Bischoff et al. [15] were weighted differently. For example, the study by Brandt et al. [18] with 28,633 patients was assigned a weight of 16%, the study by Dale et al. [32] with 93,958 patients a weight of 17.1% and the study by Hooper et al. [29] with 51,485 patients a weight of 10.1%. These weightings are not plausible. For total hip arthroplasty data, meta-analysis yielded an OR of 1.29 and for total knee arthroplasties of 1.08 – neither is significant despite the enormous sample sizes. For the meta-analysis of non-bone operations, the OR calculated was 0.75 (not significant) in favor of LAF. In the discussion the authors elaborated “[…] it seems that LAF does not reduce the risk of overall SSIs […]” for these operations (i.e., the non-bone operations) – the opposite is the case based on their meta-analysis.

Still more interesting are the last sentences in the article, stating:

“Very low-quality evidence suggests that compared with conventional ventilation, LAF ventilation does not reduce the risk of deep SSI after total hip and knee arthroplasties. Inadequate evidence suggests that LAF does not reduce the overall SSI when compared with conventional ventilation after abdominal and open vascular surgery. Conventional operating room ventilation systems appear to provide air that is clean enough for procedures involving orthopaedic implants. Given the available evidence shown by this systematic review and the previous cost-effectiveness analyses – which found LAF systems to be more expensive than conventional ventilation systems – the surgical team, infection prevention and control professionals, hospital administrators, and policy makers should not install laminar airflow equipment in new operating rooms.”

All meta-analyses identified non-significant results which the authors, on one occasion, evaluated as being of “very low-quality evidence” and, in another instance, as “inadequate evidence”. 

Furthermore, it is noteworthy that the authors themselves graded their “evidence” as being of low quality or even classified it as inadequate but, nonetheless, concluded that LAF should be rejected. The reasons for that were the costs, which tilted the balance against LAF. The authors themselves cited one study by Kramer et al. which calculated additional costs of €3.24 per procedure when using LAF. Hence, LAF whose negative effects were not substantiated by the literature review was rejected because of a cost advantage of €3 per patient. Other indisputably positive technological features of LAF (personnel protection) were not taken into consideration.

A letter to the editor on the publication by Bischoff et al. [15] was submitted by a Dutch group of authors who cast doubt on the reliability of the responses in the questionnaire. They, too, stated that medical personnel were generally not capable of stating the correct type of ventilation in use, and that furthermore, data from arthroplasty registries, likewise cited in the publication by Bischoff et al. [15], underestimated the incidence of SSIs by up to 40%. Hence, in the Netherlands, orthopaedists would continue to use LAF [37]. By way of response, the Bischoff et al. [38] authors acknowledged that, indeed, many experimental studies had shown that LAF reduced bacterial and particulate contamination of the air. However, the causal link between microbial air contamination and SSIs had not been demonstrated to date [38].

The WHO recommendation [17] also elicited comments from a German group of authors [39], who stated that LAF with ceiling panels of appropriate dimensions should be the ventilation of choice until such time as better findings were available. Evaluations in OTs had shown better results of LAF in terms of protecting staff and patients against microorganisms and surgical smoke [39].

## Differences in HVAC ceiling panels between Germany and the USA – non-comparability

Based on the experience of the authors of this paper, LAF is understood in a different context in the USA than in Germany, and apparently perceptions also differ within the various European countries. While in Germany the third filtration stage is a terminal fitting, i.e., it is installed in the operating room ceiling, that is not necessarily the case in the USA, where the third filtration stage may also be fitted in the main unit (personal communication from Candice Friedmann, Ann Arbor, Michigan, USA, and Frank Wille, Münster, Germany, 2018) or may not be installed at all [40]. That clearly demonstrates, at least when comparing Germany and the USA, that there are different variants and perceptions of LAF systems, not to mention the size of the ceiling panels. Hence, epidemiological studies from countries that do not consider that are not comparable. 

It is also thought that there are no uniform regulations and concepts on HVAC systems or ceiling panels in other countries (Korea, see above), so that a comparison of international studies is only possible if the design of the LAF system in use is precisely stated. For evaluation, the following factors are important: the number of filtration stages, filter type, configuration of the filtration stages, ceiling size, air quantities, airflow stabilizers, surgical lighting, type and extent of qualification. 

## HVAC systems with LAF reduce pathogens and particulate contamination

Myriad studies have demonstrated that bacteria and particles are considerably reduced by LAF [41], [42], [43]. In particular, surgical smoke, which may contain carcinogenic substances (pyrolysis products) and viruses (papilloma viruses) [44], [45], is rapidly eliminated [42], [46], [47], [48].It was also shown that exposure to cytostatics during modern intraperitoneal pressure aerosol chemotherapy is reduced under LAF [49].

Section 4 of the German Occupational Health and Safety Act (*ArbSchG*) stipulates that occupational health and safety measures shall be taken in accordance with the state of the art and the provisions of occupational medicine and hygiene/infection control. Moreover, Section 4 of *ArbSchG* states that personal and organizational protective measures shall be subordinated to technical measures. Section 5 of *ArbSchG* calls for hazard assessment, including the effects of biological substances. Since LAF has been shown to reduce the hazards faced by personnel from pathogens and carcinogenic surgical smoke, it is a technical measure which can be chosen in primary prevention of occupational risks for staff in OTs.

Furthermore, another study demonstrated that operating room traffic (including the number of persons present and number of times the door is opened) increased aerosolized particles and that this could be greatly reduced with LAF [50].

## Evidence of airborne infection transmission

In general, it is difficult to conclusively attribute the cause of postoperative SSI to an airborne transmission route. That is because the majority of SSI causative agents are “everyday microorganisms” that can be spread through different channels. One exception is extremely rare pathogens, for which other transmission routes can be excluded. 

Such is the case for infections caused by *Mycobacterium chimaera*, whose only portal of entry into the body is the preceding surgical procedure. Case studies have reported on contaminated heater-cooler systems used for cardiopulmonary bypass (e.g., [51], [52], [53], [54]). That case eminently demonstrates that infections can, indeed, be imputed to airborne transmission. 

Likewise, there was a report of an airborne *Trichoderma longibrachiatum* outbreak of SSIs from a defective stool/armchair [55].

## Critical limitations of LAF systems

Two aspects of LAF ventilation are very important:

- The size of the LAF ceiling and the resultant area of protection
- Positioning of instrument tables

Today, the number of instrument tables used in many operations is so great that they cannot all fit beneath a LAF ceiling measuring 3.2x3.2 m. Benen et al. [56] demonstrated that those instruments exposed outside the Class Ia ceiling area of protection, especially in Class Ib operating rooms, have higher microbial counts after a certain exposure time than instruments inside the area of protection. This also demonstrates that LAF ceilings contribute to infection protection. SSIs can also originate from unsterile instruments and implants exposed for a long period of time outside the area of protection afforded by the LAF ceiling, or from instruments that have been recontaminated (e.g., [57], [58]). To ensure the absence of microorganisms before use, instruments are cleaned, disinfected and sterilized using complex, validated processes. It is therefore important that both the surgical field and as many instrument tables as possible are placed within the LAF ceiling area of protection. Often, operating room personnel do not realize this, hence there is a need to foster greater awareness of that issue.

## Practical implications are not limited to outcome studies

Bischoff et al. [38] state that outcome studies were unable to furnish proof of an added value of LAF ventilation and concluded that LAF should not be installed. At the same time, they acknowledge that pathogens and particulate contamination can be reduced with LAF, but they disregard this with respect to infection protection and occupational health and safety.

This approach, as such, is not scientifically plausible:

- Outcome studies in the hygiene/infection control setting (see above) are in general of a low quality because of their methodology. In contrast, physical measurements and microbial count measurements (surrogate studies), for example, have very small margins of error.
- In principle, evaluations should be based on epidemiological studies, microbiology tests or experimental investigations, possibly underpinned by theoretical and logical considerations [59], [60].
- Such scientific insights are available for LAF, i.e., there is proof that it can reduce both airborne pathogens and carcinogenic surgical smoke. That, in turn, prevents microbial contamination of instruments exposed and uncovered for a long period of time on the instrument table. Furthermore, this restricts pathogen entry into open wounds.
- Likewise, it limits staff exposure to carcinogens in the nasopharyngeal region. In the interest of occupational health and safety alone, LAF ventilation must therefore be seen in a positive light [61].

## When do postoperative SSIs occur?

*KRINKO* [62] reports that primary wound healing closure without drainage is generally seen after 24 hours and the wound is no longer deemed susceptible to exogenous infection. Therefore the wound dressing can be removed after 24 to 48 hours. 

Likewise, during primary wound healing, the wound is at risk for endogenous SSIs through haematogenous seeding of bacteria. 

It can be inferred that most exogenous postoperative SSIs are causally linked to the time spent in the operating room and less to the postoperative care, e.g., in the patient’s room [63], [64], [65].

One possible cause may be inadequate preoperative skin antisepsis, especially given that bacteria in the hair follicles may not have been effectively inactivated [66].

It is also well known that implantation of foreign bodies presents a special risk of SSI [61]. It has been demonstrated that the minimum infectious dose required for *Staphylococcus aureus* abscess formation was 10,000–100,000 times lower when a foreign body was implanted [67], [68]. It has been reported that colonization of foreign bodies with even 100 pathogens can trigger infection [61].

From this it can be concluded that the majority of SSIs are contracted during the time spent in the operating room [69] and that implants present a particular risk. Apart from the surgical procedure itself, risks emanate from instruments contaminated with airborne microbes, bacterial shedding from surgical staff as well as inadequate preoperative disinfection, especially in the region of the hair follicles. Charnley already reported that 50 years ago [4], [70], [71].

## The influence of poor discipline in the operating room

It has been demonstrated that poor discipline of surgical staff increases the SSI risk [72]. Already for several years now, the *KRINKO* Recommendation for Prevention of Postoperative SSIs has stipulated that the protective headgear and oronasal mask must completely cover all beard and head hair as well as the mouth and nose [62], [73]. Likewise, the standard requirement specified in the literature is that all hair must be fully covered [61]. 

Table 2 (Tab. 2) illustrates bacterial shedding from the body under different conditions.

In certain cases it has been possible to attribute SSI outbreaks to individual surgeons who were carriers [74].

It has been demonstrated that of the parts of the head that (may have) remained uncovered during the operation, the ears were responsible for most bacterial shedding, i.e., three times more than the forehead or the eyebrows [75]. Besides, it must be noted that in many operations, the surgeon’s head is very close to the surgical site, often for prolonged periods of time. An earlier study drew attention to the importance of wearing proper headgear, and demonstrated a massive rise in bacterial shedding upon omission of headgear [76].

The *KRINKO* requirement that the protective headgear and oronasal mask must completely cover all beard and head hair as well as the mouth and nose can only be fulfilled by wearing an Astro helmet/hood, which together with a properly worn oronasal mask, will cover even an extensive beard. However, it must be ensured that the surgical helmets are of a high standard with regard to particle retention [77].

The current real-life situation: Often, the hair is uncovered, ears are not covered and even in the case of staff at the operating table, the mask frequently does not fit tightly.

## Skin diseases are an additional risk

The *KRINKO Recommendation for Hand Hygiene* [78] was the first to draw attention to the problem of chronic skin diseases, suggesting that if necessary, through the intervention of the occupational physician, colonization with potential pathogens should be investigated and eradication attempted. After all, atopic eczema and psoriasis are both seen in around three percent of adults. More attention must be paid to that problem in the future in the operating room.

- Operating room personnel should be tested for critical bacterial colonization in case the staff is suspected of being the source of postoperative wound infections. If critical colonization is found, efforts should be made to eradicate highly pathogenic bacteria (MRSA – currently not possible for MRGN and VRE, possibly with the exception of *Acinetobacter* on skin).
- Risk assessment must be conducted and, if necessary, critical skin sites covered.

## Operating rooms as clean rooms

On 1 August 2007, the Human Tissue Regulations (Quality and Safety of Human Tissues and Cells) [79] came into force in Germany, transposing into national law Directive 2004/23/EC of the European Union from 2004. Since then, tissue preparations, which Section 1a(4) of the Transplantation Act considers to be tissues or matter prepared from tissues, are defined as medicinal products pursuant to Section 4(30) of the German Medicinal Products Act (*AMG*). Tissue preparations include human corneas, human amniotic membranes, skin, cardiovascular tissue such as cardiac valves and blood vessels, as well as musculoskeletal tissues such as femoral heads and bone preparations, soft tissues (fascia and tendons) and bone cartilage. 

With the new directive, there are now uniform quality and safety standards throughout Europe for the donation, procurement, testing, processing, preservation, storage and distribution of human tissues and cells [80]. When handling and processing tissues in a tissue bank, air quality that minimizes the risk of microbial contamination must be assured. The environment must meet at least class A cleanliness and the background environment class D cleanliness of the EC guide to Good Manufacturing Practice (EU GMP). In Germany, pursuant to Section 64 of *AMG*, tissue banks are inspected by the responsible state authorities at least once every two years after being licenced. Increasingly, the state authorities apply the same requirements for tissue procurement as for tissue processing. As such, operating rooms become class A clean rooms, which implies that in operating rooms where tissue procurement/harvesting takes place, LAF is needed. 

## Conclusions

The following conclusions can be drawn from the above:

- The publications by Bischoff et al. [15] and Allegranzi et al. [16] report on various studies which for methodological reasons cannot be used for evaluation of LAF ventilation. These include the studies by Hooper et al. [29] (2011 – implausible infection rates), Namba et al. [31] (2012 – USA: other LAF standards), Miner et al. [35] (2007 – USA, other LAF standards) and Jeong et al. [34] (2013 – probably other LAF standards in Korea). Likewise, the studies by Brandt et al. [18] and Breier et al. [25] from Germany have obvious methodological shortcomings. That leaves very few studies, some of which demonstrate the protective effect of LAF but, overall, do not suffice to permit definitive evaluation.
- There is no doubt that LAF is better at reducing bacteria and particles than conventional turbulent mixed airflow and that it also removes carcinogenic smoke more effectively. That contributes to personnel protection and corresponds to the perception in Germany of the primacy of primary protection (technical protective measures) in the workplace to the extent possible, as in this case.
- Since LAF reduces bacteria and particles, it can help to restrict pathogen entry into the surgical site. This is of particular relevance for long operating times.
- Accordingly, LAF confers benefits in operating rooms.
- Likewise, DIN 1946-4 [14] continues to feature class Ia rooms (LAF) and, as such, LAF reflects the state of the art to be implemented in hospital construction in Germany. Therefore, at least some of the operating rooms in newly built hospitals should be equipped with LAF ceilings.
- Because of the growing trend towards tissue procurement and associated requirements, it can be assumed that LAF will be mandatory in future and that the requirements governing the operating room will be upgraded to those of clean rooms.
- The possible causes of SSIs include:
  - Gaps in preoperative skin antisepsis, e.g., bacteria in the hair follicles not effectively inactivated.
  - Bacterial shedding in dead skin and hair from the heads of surgical personnel.
  - Aerosols from the nasopharyngeal region. Hence, the quality and correct fit of oronasal masks play a crucial role.
  - Contaminated instruments, e.g., those exposed outside the area of protection of the LAF ceiling where they become recontaminated.
  - The surgeon’s hands, if gloves are damaged or have manufacturing defects.
  - Airborne pathogens (adhering to particles) subjected to turbulence.
  - Haematogenous seeding of bacteria following interventions conducive to bacteraemia.
- Of the regions of the head often left uncovered during an operation in the operating room, the ears are responsible for most bacterial shedding. This means that the ears, too, must definitely be covered with a cap during an operation. The same applies for all beard and head hair. Astro caps/hoods in conjunction with tight fitting oronasal masks are the only solution for complete coverage of hair, beard and ears. However, attention must be paid to the quality of the helmets, since particle penetration through thin caps may be easier. The hospital’s medical superintendent, nursing directors, heads of surgical departments and operating room management are responsible for implementing an appropriate professional dress code. That, above all, implies acting as role models.

In summary, it is impossible to issue a recommendation against the use of LAF ventilation in the operating room. LAF ceilings assuring an area of protection of 3.2x3.2 m are superior to conventional turbulent ventilation – they are more effective at reducing bacteria and particles and at removing potential carcinogenic smoke, thus protecting patients, surgeons and exposed instruments. Therefore, as stipulated by the currently valid DIN 1946-4, LAF ventilation should be installed in surgical departments due to the risk of the surgical procedures conducted there.

## Notes

### Competing interests

The authors declare that they have no competing interests.

## Figures and Tables

**Table 1:**
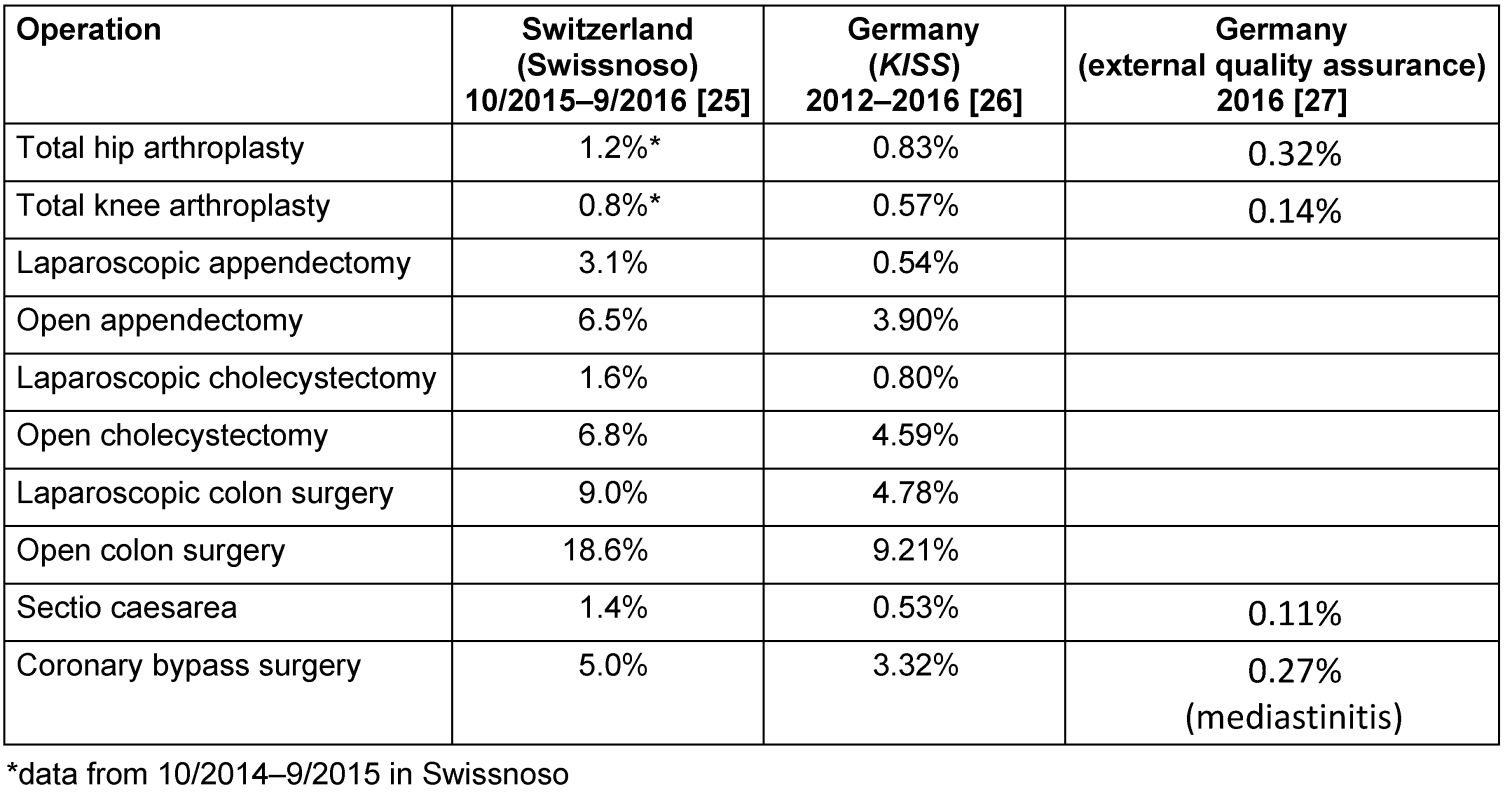
Comparison of HAI rates using different infection recording systems

**Table 2:**
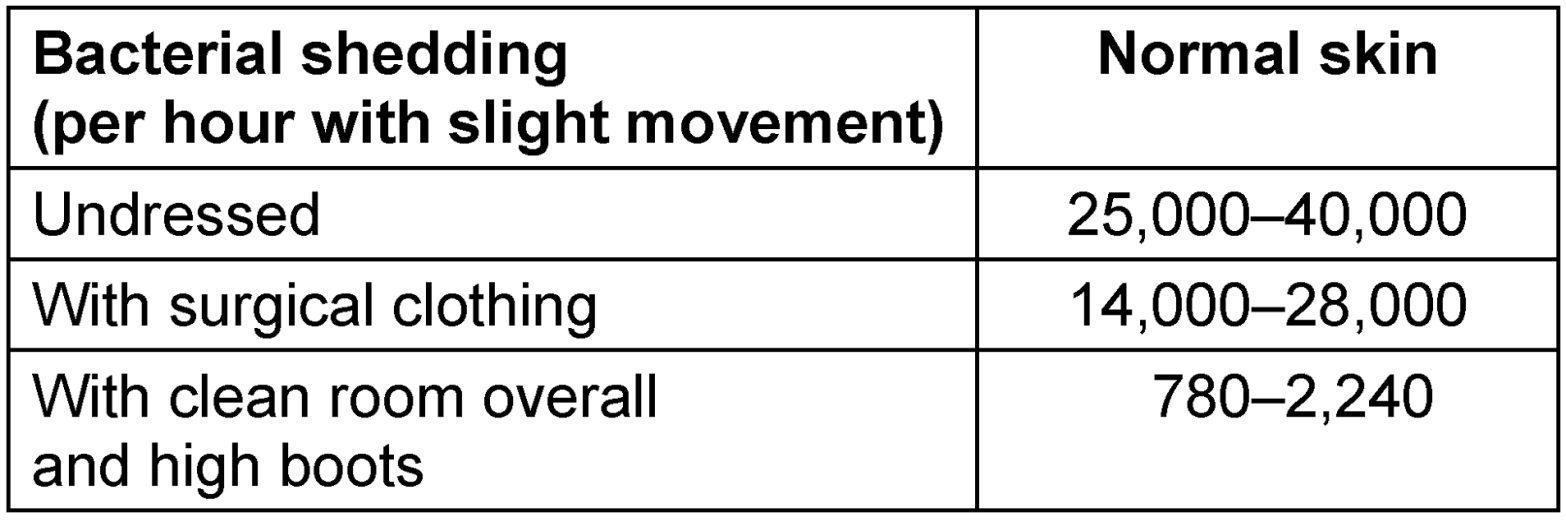
Bacterial shedding in relation to different types of clothing [based on 84]
